# mtDNA-Server 2: advancing mitochondrial DNA analysis through highly parallelized data processing and interactive analytics

**DOI:** 10.1093/nar/gkae296

**Published:** 2024-05-06

**Authors:** Hansi Weissensteiner, Lukas Forer, Florian Kronenberg, Sebastian Schönherr

**Affiliations:** Institute of Genetic Epidemiology, Medical University of Innsbruck, Innsbruck, Austria; Institute of Genetic Epidemiology, Medical University of Innsbruck, Innsbruck, Austria; Institute of Genetic Epidemiology, Medical University of Innsbruck, Innsbruck, Austria; Institute of Genetic Epidemiology, Medical University of Innsbruck, Innsbruck, Austria

## Abstract

Over the past decade, mtDNA-Server established itself as one of the most widely used variant calling web-services for human mitochondrial genomes. The service accepts sequencing data in BAM format and returns an annotated variant analysis report for both homoplasmic and heteroplasmic variants. In this work we present mtDNA-Server 2, which includes several new features highly requested by the community. Most importantly, it includes (a) the integration of a novel variant calling mode that accurately call insertions, deletions and single nucleotide variants at once, (b) the integration of additional quality control and input validation modules, (c) a method to estimate the required coverage to minimize false positives and (d) an interactive analytics dashboard. Furthermore, we migrated the complete analysis workflow to the Nextflow workflow manager for improved parallelization, reproducibility and local execution. Recognizing the importance of insertions and deletions as well as offering novel quality control, validation and reporting features, mtDNA-Server 2 provides researchers and clinicians a new state-of-the-art analysis platform for interpreting mitochondrial genomes. mtDNA-Server 2 is available via mitoverse, our analysis platform that offers a centralized place for mtDNA analysis in the cloud. The web-service, source code and its documentation are freely accessible at https://mitoverse.i-med.ac.at.

## Graphical Abstract

**Graphical Abstract:**
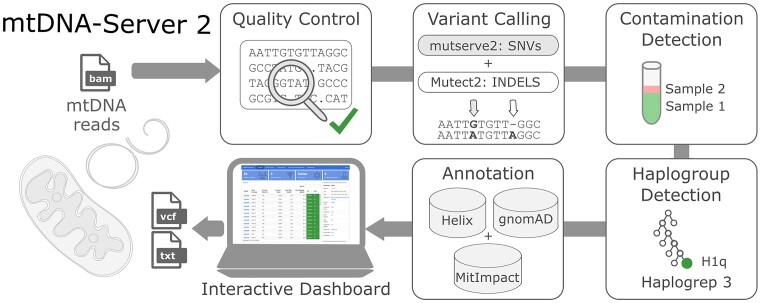

## Introduction

Mitochondria, mainly involved in energy production, are highly dynamic organelles that regulate and participate in complex cellular processes. Mitochondria possess their own DNA, known as mitochondrial DNA (mtDNA), which encodes a subset of essential oxidative phosphorylation proteins, transfer RNAs and ribosomal RNAs (1). Mitochondrial DNA exists in multiple copies within each mitochondrion and cell which can accumulate to different mitochondrial haplotypes. The mixture of mitochondrial alleles within one individual is denoted as heteroplasmy and has been attributed to ageing and several diseases (2–4). The correct estimation of heteroplasmic levels is not only dependent on the tissue of interest due to its varying copy number or wet-lab protocols but mainly on an accurate and reproducible bioinformatics analysis (5). For this purpose, we have previously developed mtDNA-Server, a graphically cloud platform to detect and annotate heteroplasmic and homoplasmic variants. mtDNA-Server has been widely adopted by the community and it has been shown that it performs well on large datasets and can accurately call heteroplasmic variants on single nucleotide variants (SNVs) (6). We also improved the service over the last years by integrating new statistical models to the underlying variant caller mutserve, which shows accurate results for mtDNA heteroplasmy analysis (7). Due to the fast and accurate variant calling process, mutserve has also been integrated into other pipelines for SNV detection (8). However, its lacking support for insertions and deletions (INDELs) combined with new requirements for large next-generation or third-generation sequencing datasets, spurred the need for further development or enhancement of the platform.

Here, we present the updated web-service mtDNA-Server 2, which integrates several new and improved data analysis steps to support users during analysis. First, it integrates our recently published approaches on haplogroup classification (9) and contamination detection (10) in combination with the improved mutserve2 variant caller. Furthermore, it includes a new quality-control and input validation module to detect errors early in the analysis process and provides users feedback in a standardized way. Most importantly, mtDNA-Server 2 offers a new variant calling mode (so called fusion mode) that accurately combines INDELs from GATK Mutect2 with SNVs from mutserve2. We also added an analysis module that estimates the required coverage for a detected variant allele frequency (VAF) using a binomial distribution approach (11), which minimizes the number of reported false positives due to low coverage. To allow researchers and clinicians a comprehensive view on the data, we also offer a new interactive analytics dashboard. This dashboard links from a summary to a detailed view of each sample, whereas each variant is annotated with information from mitochondrial databases. Besides the developed web-service, mtDNA-Server 2 is provided as a Nextflow DSL2 workflow, which will help users to analyze their data also on local workstations, local clusters or cloud infrastructures (e.g. Research Analysis Platform from UK Biobank). Overall, we present a best-practice web-service for the analysis of mitochondrial genomes and show that the service can accurately call variants in a reproducible and fast way, bringing mitochondrial analysis closer to clinical utility.

## Materials and methods

### Design and implementation

The mtDNA-Server 2 pipeline is implemented as a Nextflow DSL2 pipeline (12) and uses the Cloudgene platform (13) to offer it as a web-service to the community. The pipeline is parallelized on a BAM file level and consists of several independent modules for data analysis. All necessary software dependencies utilized by the pipeline are available in containers, offering portability and facilitating the local execution. This modular architecture of mtDNA-Server 2 allows an easy integration of future improvements (e.g. new variant calling tools or additional annotations) using a well-accepted workflow manager. We also applied the novel nf-test framework for Nextflow pipelines to ensure that the pipeline code works correctly, which also improves its robustness and maintainability against future updates. We integrated the Nextflow workflow into Cloudgene, a robust and secure framework that offers user authentication, job submission including queuing and download or sharing of results. All results can be analyzed directly in the browser and unique links shared with collaborators.

### Input validation and quality control

To support users and identify problems early in the analysis process, we developed an input validation and quality-control module. This module validates input parameters, analyzes read and mapping quality as well as the coverage of uploaded samples and excludes individual samples if requirements such as coverage or correct mtDNA contigs are not fulfilled. The number of passed samples, all set input parameters and quality control statistics are reported back to users. For detailed QC analysis, users can access individual FASTQC reports which are provided as a combined MultiQC report (14).

### SNV and INDEL variant calling

**Figure 1.**
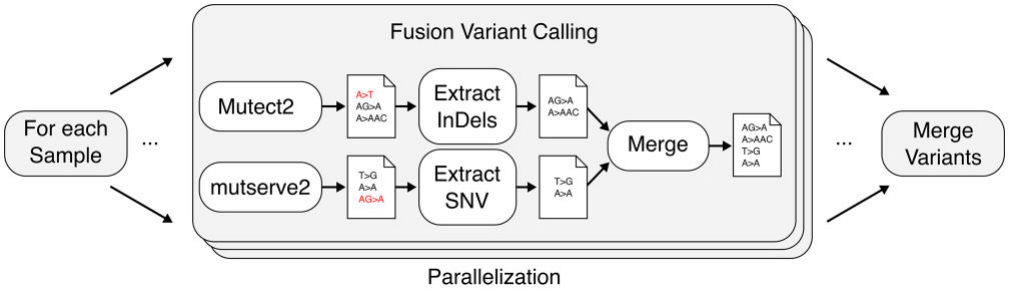
Improvement of Variant Calling. The fusion variant calling mode combines the output of Mutect2 with mutserve2. For each sample, both variant callers are executed in parallel, and variants are reported in VCF format. After normalizing INDELs and splitting multiallelic sites, we apply a merging tool that combines the Mutect2 output with the output from mutserve2. If both variant callers report a variant on the same position, the INDEL variant is favored. The merged variants are annotated and reported back to the user.

Previously, we developed the mutserve variant caller (15) and integrated it into mtDNA-Server. Originally developed for SNV detection, several comparisons show that mutserve performs well especially on large datasets (6) and has also been integrated into other pipelines (8). We have now migrated it to a stand-alone tool (mutserve2) and integrated several new features such as a (a) variant annotation, (b) integration of a Bayes model for variant calling and (c) VCF support. Mutserve2 includes the Bayes approach to define heteroplasmy by accounting for the sequencing error and determine true positive heteroplasmy (16). Using a Bayes model, we combine the prior probability (using variant frequencies from the 1000 Genomes Project) and the likelihood (from the base quality) to calculate the posterior probability of each genotype (A,C,G,T) and finally select the genotype with the highest probability. The underlying model of mutserve2 allows calling especially low-level heteroplasmic sites (up to 2%) with high accuracy (7,17) and outpaces other variant callers in speed. mutserve2 is used for SNV calling within mtDNA-Server 2. For INDEL calling, mtDNA-Server 2 integrated the GATK Mutect2 variant caller. Besides the Mutect2-only and mutserve2-only modes, we integrated a new fusion mode that combines the advantages of both variant callers (see Figure 1). Within the fusion mode, both variant callers are first executed and results in VCF format are then normalized (for INDELs) as well as multiallelic positions are split. A novel developed tool combines the results of both variant callers by using INDELs from Mutect2 and SNVs from mutserve2. If both variant callers include overlapping results (i.e. INDEL and SNV at the same position), Mutect2 calls are ranked higher. The final call set is then annotated and returned to the user. The mutserve2 results are further used for haplogroup estimation with Haplogrep 3 and contamination detection with haplocheck.

### Coverage estimation and subsampling

Variant calling includes models to minimize the number of false positives and false negatives. Especially for variants with clinical impacts, confident and reproducible detection for variant allele frequencies is crucial. To account for this, we integrated a novel method based on the binomial probability distribution presented by Petrackova *et al.* (11) to determine the minimal trusted variant allele frequency (VAF) level by using the mean base quality and coverage of a specific variant. We ported the available code to Java and incorporated it as an optional module into the workflow. For each variant detected by mutserve2, the required VAF is calculated, and variants are flagged in the output in case the statistical requirements are not fulfilled. To report possible false negatives, mtDNA-Server 2 also calculates a mean level of detectable VAF for each sample which is reported within the input validation module. Using the recommendations from (11), we also set a default level of 2% for heteroplasmy detection within mtDNA-Server 2, which can be adapted by the user. Besides the coverage estimation module, mtDNA-Server 2 also provides a new optional input parameter to subsample input BAMs to a coverage of 2000x. Using the mean coverage of each sample, we calculate the fraction of required reads and use the samtools package for subsampling. To generate reproducible results, we use a static seed value for each subsampling process.

### Evaluation and validation

To evaluate mtDNA-Server 2, we used the publicly available dataset including 30 samples (coverage 100x and 2000x) from the previously published mitoHPC pipeline (8). This dataset includes SNVs and INDELs and shows the pitfalls of the previously published mtDNA-Server workflow regarding INDELs. Additionally, we also run a subset of 10, 20 and 50 samples from the 1000 Genomes Project (18) on mitoverse to show the scalability and its report functionality. To test the stand-alone Nextflow workflow on the command-line, we have also run the complete set of 1000 Genomes Project consisting of 2504 samples and 162 GB input data on a single node (12 CPUs).

## Results

### Web-service

**Figure 2.**
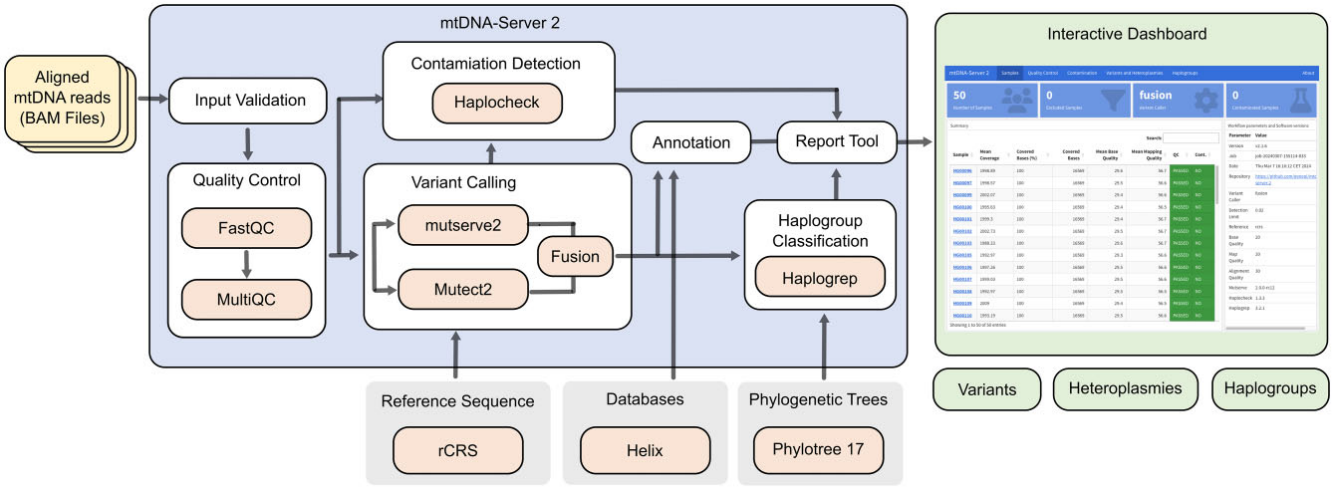
mtDNA-Server 2 Workflow. First, users upload BAM files, and an input validation step is executed. For all samples, which are passing input validation, a FastQC report is created, summarized with MultiQC. Next, one of the available variant callers (mutserve2, Mutect2 or fusion) can be selected. For the fusion mode, results of Mutect2 (InDels) and mutserve2 (SNVs) are merged. Detected variants are annotated, a check for contamination and a haplogroup assignment process are executed. MtDNA-Server also provides an optional coverage estimation step especially for clinical practice, to verify that coverage is sufficient for the detected variants given the mean per-base qualities. All created files are finally summarized in an interactive HTML report including links to sample-specific reports.

mtDNA-Server 2 has been integrated into our mitoverse platform, which offers a centralized place for mtDNA analysis in the cloud. Besides the already available applications (haplocheck and Haplogrep3), users can now select the new mtDNA-Server 2 application. Initially, users must register with a username and an optional email address to start their analysis. After selecting the BAM data, several input parameters such as the VAF detection limit, quality values or coverage estimation need to be specified. Most importantly, users can choose between (a) accurate SNV calling with mutserve2 which offers the fastest computational performance, (b) Mutect2 SNV and INDEL calling or (c) a fusion mode that combines the strength of both methods by considering INDELs from Mutect2 and SNVs from mutserve2. Uploaded data are added to a server processing queue and as soon as the job is running, users receive quality control (QC) statistics. If the coverage subsampling option has been enabled, BAMs are subsampled before QC statistics are generated. For all samples that passed the input validation step, the variant calling process is started. In case no samples passed this step, users can still analyze the QC report and get detailed information on the job failure. If the coverage estimation parameter has been selected, mtDNA-Server 2 verifies if the detected coverage is sufficient to reliable report a variant. Subsequently, mitochondrial haplogroup classification using Haplogrep 3 and contamination detection using haplocheck is executed on the final variants file. Finally, variants are annotated, all generated data is summarized in a summary dashboard including links to individual sample reports (see Figure 2). In case an email address has been registered, users are notified via email on the job status. Input data is deleted right after the analysis, results are available on the server for 7 days.

### Interactive analysis dashboard

**Figure 3.**
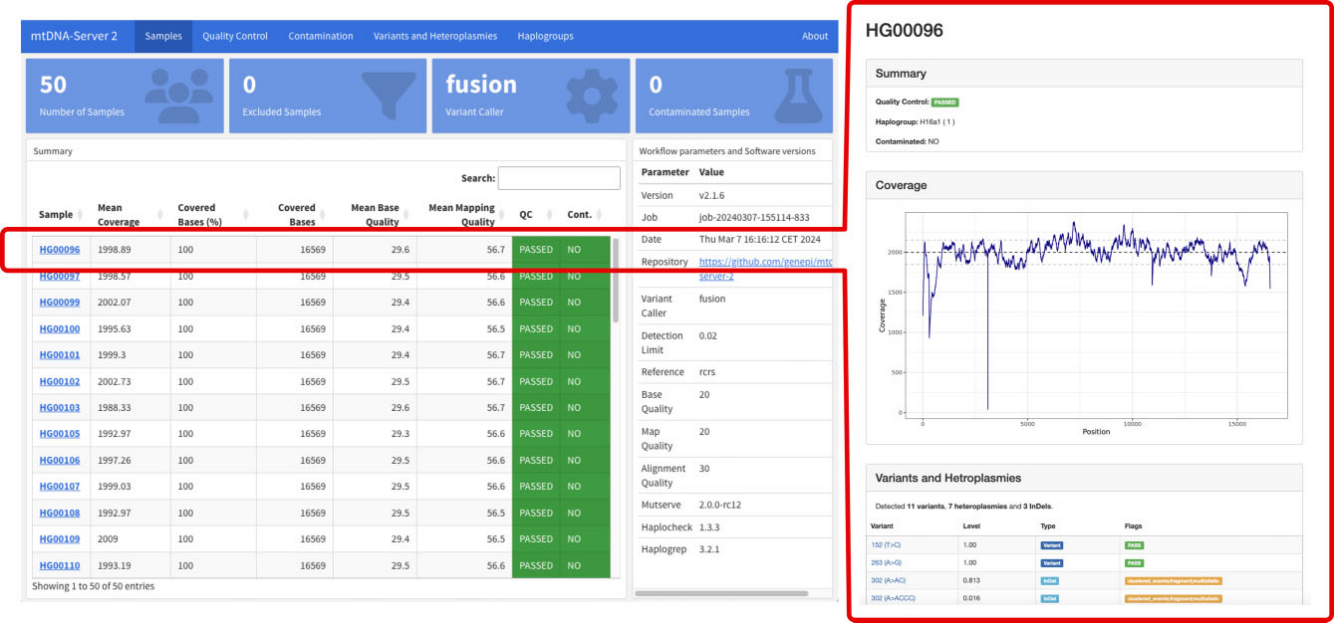
Interactive Analytics Report. The report includes a summary over all samples including quality control statistics, contamination status and applied parameters. Each sample is directly linked to an in-depth sample report. The sample report gives an overview over all statistics but also include a coverage plot and a list of all detected SNVs and INDEL positions with a rich set of annotations from mitochondrial databases (not shown in the figure).

A comprehensive overview of the analyzed data is key for downstream analysis. Therefore, we integrated a new analysis dashboard for data visualization consisting of (a) a summary report including quality-control statistics and the status of each sample and (b) a detailed sample report including a variants list or coverage reports (see Figure 3). The summary report consists of a list of all analyzed samples. It includes a table of all passed and filtered samples, mean coverage information, quality values and contamination status. This provides users with a quick overview of all samples and helps them to identify outliers or excluded samples. Additional tabs allow users to navigate through contamination results, haplogroups and summary statistics on SNVs and INDELs. It also includes the selected input parameters to reproduce the results. By linking the overall report to individual sample reports, users can navigate through the data in an interactive way. The sample report integrates all detected variants which are linked to Haplogrep 3 and includes annotations from various publicly available genome databases such as gnomAD v3.1 (6). Besides the graphical annotation, downloadable variants are annotated using the mutserve2 annotation command providing information on the locus, scores, amino acids, NUMTs (19) and information from the Helix mitochondrial database (20) and Mitimpact (21,22). The sample report also includes information on parameters, quality metrics and contamination status and, most importantly, a list of all detected SNVs and INDELs and coverage reports. These reports can also be shared with collaborators via a unique link and are accessible without login or registration.

### Validation and accuracy

To validate the new variant calling modes, we re-analyzed 30 simulated samples from Battle *et al.* (8) with two different coverage depths (100× and 2000×). Each of the samples represents a main haplogroup with 43 introduced heteroplasmic sites (8 INDELs, 35 SNVs). Battle *et al.* included in their analysis also the results of the previous version of mitoverse (using mtDNA-Server). We have reanalyzed the data by uploading the dataset directly to mitoverse using mtDNA-Server 2 and compared it to their previous results. The newly developed fusion mode shows the best performance and detects only a minimal number of false positives and negatives over all 30 samples for both coverage experiments (see Table 1). While the new version of mtDNA-Server correctly detects almost 100% of the 43 variants, the previous version only detected 36.97 variants (100×) and 39.17 (2000×) (see (8)). Compared to the mitoHPC pipeline, a slightly lower result for the 2000× data (43.1 for mtDNA-Server 2 versus 43.0 for mitoHPC) has been reached. We speculate that this is due to an additional step within mitoHPC, where samples are aligned against its own consensus sequence as reference which also results in higher computational costs (see Discussion).

**Table 1.** Average number of heteroplasmic sites identified across simulated data from (8)

	SNV + InDel			SNV			InDel		
	All sites	False negatives	False positives	All sites	False negatives	False positives	All sites	False negatives	False positives
**100× coverage**									
mutserve2	35.43	8.1	0.47	35.43	0.1	0.47	0	8	0
Mutect2	43.63	1.3	1.87	35.47	0.4	0.8	8.17	0.9	1.07
Fusion	43.1	1	1.1	34.93	0.1	0.03	8.17	0.9	1.07
**2000× coverage**									
mutserve2	35.6	8	0.6	35.6	0	0.6	0	8	0
Mutect2	43.47	0.5	0.93	35.47	0.47	0.9	8	0.03	0.03
Fusion	43	0.07	0.1	35	0.03	0.07	8	0.03	0.03

In total 43 variants (35 SNVs, 8 InDels) are included in the gold standard. The fusion mode shows the best overall performance. In the 100x coverage 1 false positives and 3 false negatives are detected over all samples. In the 2000× coverage, 2 false positives and 1 false negative variant are detected over all 30 samples.

### Scalability and runtime

We also tested a subset of the 1000 Genomes Project deep coverage data (18) on the mitoverse platform (10, 20 and 50 samples). The sample mean coverage was between 10 000×–27 000× and the runtime scales linear with the number of samples. For 50 samples (3.2 GB), the analysis applying the fusion mode (SNVs + INDELs) required 4 h 20 min (34 CPU hours), whereas the mutserve2 mode (SNVs-only) required 1h 42 min (14 CPU hours). The runtime can be significantly lowered by applying the available subsampling feature of mtDNA-Server 2. Applying the available subsampling, reduced the runtime for 50 samples (using the fusion mode) from 4 h 20 min to 26 min (4.6 CPUs) but still showing identical results.

### mtDNA-Server 2 as a stand-alone tool

Nextflow allows users to develop multi-step workflows which can be run locally, on clouds or on cluster computing environments. Due to the integrated container technology, extensive configuration and installation is avoided, and a workflow is portable between systems. This enables users to run the mtDNA-Server 2 workflow on the command-line in a local and secured environment. We executed the 1000 Genomes Phase 3 data (*n* = 2504) on a local system with 12 CPUs. Using the SNV mode (mutserve2), we were able to process this large data set data in 12 h (141.5 CPU hours). Due to the separation of the summary and view, users can navigate the reports of this large dataset in an interactive and fast way.

## Discussion

Many studies highlighted the importance of mitochondrial genomes and their role for diseases and within the ageing process. Recent studies on the currently largest available datasets from UK Biobank and AllOfUs (23) further showed that nearly every human harbors heteroplasmic mtDNA variants and especially investigated the crucial role of heteroplasmic INDELs (2). With the development of mtDNA-Server 2, we provide a scalable and reproducible workflow that includes several important analysis steps necessary to detect homoplasmic and heteroplasmic SNVs and INDELs. Compared to the previous version, we added a new variant calling mode based on Mutect2 and mutserve2 that allows to jointly call SNVs and INDELs. Our results show that this mode yields the highest accuracy in combination with good computational efficiency, making it well-suited for a freely accessible web-service. Furthermore, we provide new workflows steps such as input validation and quality-control, a method to estimate the required coverage for an individual variant and offers an interactive HTML report that allows users to explore a dataset directly in the web browser starting from a broad overview to a sample-specific variant view including a rich set of annotations. The availability of mtDNA-Server 2 as a standalone Nextflow workflow will allow users to run it in a secured environment ensuring reproducible results which are identical to the web-service.

There are currently no functional web-services available for heteroplasmy detection. Over the last years, two well-designed command-line analysis pipelines (2,8) have been developed. Most interestingly, both pipelines include a mode to align data against their own consensus sequence as a reference. Since this seems to have advantages in specific situations (e.g. removing coverage drop in non-European samples), we plan to integrate this feature also within mtDNA-Server 2. Due to our module-based architecture, the integration is straightforward and requires only minor adaptations to the overall workflow. Additionally, mtDNA-Server 2 currently only annotates detected NUMT positions in the final output file. While the implemented binomial approach reduces the number of false positives resulting from low coverage, it does not decrease the number of NUMT derived false positives due to a low mitochondrial copy number (mtCN) (6). To filter NUMT positions already in the aligning process, we also plan to initially re-align samples not only to the rCRS or its own consensus sequence but also against a set of predefined NUMTs (24).

Overall, mtDNA-Server 2 improves an already well-accepted graphically cloud platform by providing several new features to the community. The pipeline now provides many long-expected features, prioritizes scalability and has undergone rigorous testing. Its modular and well-accepted architecture will allow us and others to steadily improve the pipeline over the next years.

## Data Availability

mtDNA-Server 2 is available at https://mitoverse.i-med.ac.at. This website is free and open to all users. Upload and analysis of sensitive personal information requires a login. The source code is freely available at https://github.com/genepi/mtdna-server-2 and https://doi.org/10.5281/zenodo.10931377. The documentation including example data is available at https://mitoverse.readthedocs.io.
